# α-Enolase, an Adhesion-Related Factor of *Mycoplasma bovis*

**DOI:** 10.1371/journal.pone.0038836

**Published:** 2012-06-13

**Authors:** Zhiqiang Song, Yuan Li, Yang Liu, Jiuqing Xin, Xiaohui Zou, Wenjing Sun

**Affiliations:** 1 National Contagious Bovine Pleuropneumonia Reference Laboratory, Division of Bacterial Diseases, State Key Laboratory of Veterinary Biotechnology, Harbin Veterinary Research Institute, CAAS, Harbin, China; 2 National Engineering Research Center of Veterinary Biologics, Harbin, China; Miami University, United States of America

## Abstract

*Mycoplasma bovis* is the causative agent of *Mycoplasma bovis*-associated disease (MbAD). Although the mechanisms underlying *M. bovis* adherence to host cells is not clear, recent studies have shown that the cell surface protein α-enolase facilitates bacterial invasion and dissemination in the infected host. In this study, we cloned, expressed and purified recombinant *M. bovis* α-enolase and induced polyclonal anti-α-enolase antibodies in rabbits. *M. bovis* α-enolase was detected in the cytoplasmic and membrane protein fractions by these antibodies. Triple immunofluorescence labeling combined with confocal laser scanning microscopy (CLSM) revealed that the plasminogen (Plg) enhanced the adherence of *M. bovis* to embryonic bovine lung (EBL) cells; the values obtained for adherence and inhibition are consistent with this finding. Interestingly, we found that trace amounts of trypsin acted as a more effective enhancer of cell adherence than Plg. Hence, our data indicate that surface-associated *M. bovis* α-enolase is an adhesion-related factor of *M. bovis* that contributes to adherence by binding Plg.

## Introduction

The adherence of mycoplasmas to the host cell initiates infection with bacteria of this genus [1]. *Mycoplasma bovis* is the causative agent of *Mycoplasma bovis*-associated disease (MbAD) [2]. The bacterium was first isolated from a case of severe mastitis by Hale in 1961 [3]. *M. bovis,* which causes pneumonia, otitis media and arthritis in young calves, has been an important cause of disease in North America,Europe and Asia [4]–[6]. *M. bovis* was first isolated in the Hubei province of China in 2008 [6], but the economic cost of MbAD has not been reported.

Plasminogen (Plg) is a single-chain glycoprotein (with a molecular mass of 92 kDa) that is converted into plasmin *in vivo* [7]. Many bacteria express surface structures that interact with Plg and specific receptors on their cell surfaces enhance the activation of Plg via Plg activators [8]. Many pathogens have been found to capture Plg, which allows the bacteria to acquire surface-associated proteolytic activity that may facilitate bacterial invasion and dissemination in the infected host [9].

Recently, the glycolytic enzyme α-enolase, a non-classical Plg-binding protein, has been found in many bacteria [9]–[12]. Prokaryotic α-enolase is a highly conserved protein that may contribute to pathophysiological processes [13]. Research has shown that the presence of bacterial surface α-enolase is closely related to bacterial adherence to the host cell [14]. There is, however, no evidence showing that *M. bovis* α-enolase (MbEno) is a membrane protein related to *M. bovis* adherence to the host cell.

In this study, we found that *M. bovis* expresses several plasminogen-binding proteins. We used recombinant *M. bovis* α-enolase (rMbEno) to induce anti-α-enolase antibodies in rabbits to facilitate characterization of the adherence properties of *M. bovis* to embryonic bovine lung (EBL) cells. We also explored the role of α-enolase as a Plg-binding protein in adherence and invasion of *M. bovis*.

## Results

### Identification of enolase of *M. bovis*

The 1365-bp open reading frame (ORF) of α-enolase was identified in the complete genomic sequence of *M. bovis* strain Hubei. The ORF encoded a 454-amino-acid protein with a theoretical molecular weight of 49369 Da and isoelectric point of 5.27 (Pepstats V6.0.1). The *M. bovis* α-enolase lacks classical protein-sorting signals such as N-terminal signal peptides, hydrophobic domains, or a C-terminal LPXTGX motif (SOSUI). The amino acid sequence was homologous to the α-enolase sequences from a variety of species, as determined using a maximum-likelihood analysis in MEGA4.0.2. The *M. bovis* Hubei α-enolase identified showed more than 90% homology to *M. bovis* PG45 (E4PZX0), *M. fermentans* (E1PS24), *M. agalactiae*(A5IYA8), *M. hyopneumoniae* (Q601S2) and *M. gallisepticum* (Q7NAY0), respectively. In addition, the protein contained features typical of Plg-binding-site motifs including lysine as the C-terminal residue (FYNIK), and a conserved, positively charged lysine-rich internal motif (LYDENSKKY), as identified by UniProt (data not shown).

### *M. bovis* α-enolase gene expression, and protein purification

We designed primers to mutate TGA into TGG to obtain a sequence that would be correctly expressed in *E. coli*. The recombinant plasmid was digested with restriction enzymes to verify the size of the insert DNA and that TGA (186 bp and 960 bp) had been successfully mutated into TGG.

Recombinant plasmids were transformed into *E. coli* BL21 (DE3) pLysS cells to obtain the recombinant fusion protein designated His-rMbEno. His-tagged recombinant protein, purified under non-denaturin conditions (using Ni-NTA His•Bind Resin) had an apparent molecular weight of 72 kDa.

### The α-enolase antibody

**Figure 1:**
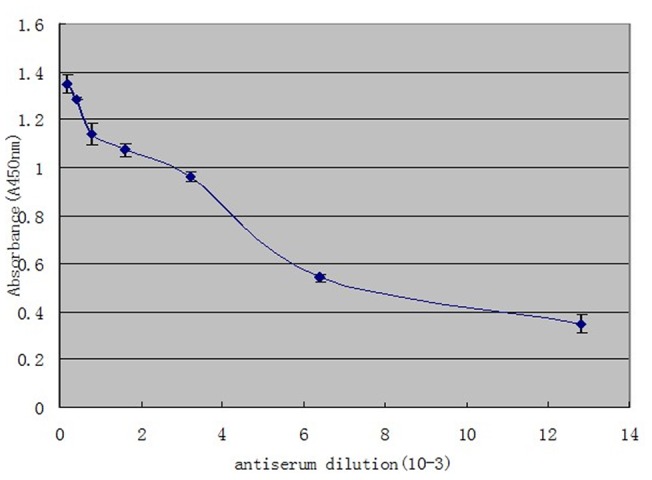
Binding of anti-α-enolase antibodies to recombinant *Mycoplasma bovis* α-enolase (rMbEno). ELISA plate wells were coated with rMbEno (1.0 ug protein/well). Well contents were reacted with serial dilutions (1/200 to 1/12800) of rabbit anti-α-enolase antibodies, followed by anti-rabbit IgG(whole molecule) peroxidase conjugate. Results were determined using o-phenylenediamine as a substrate, as described in Materials and Methods.

Ten days after the third immunization, the reactivity and specificity of the rabbit antisera was tested by enzyme-linked immunosorbent assay (ELISA) (**Figure** **1**), Following purification with Protein A sepharose, the serum, containing anti-rMbEno polyclonal antibodies (2.0 mg/ml), was stored at −20°C.

### Localization of M. bovis α-enolase

**Figure 2:**
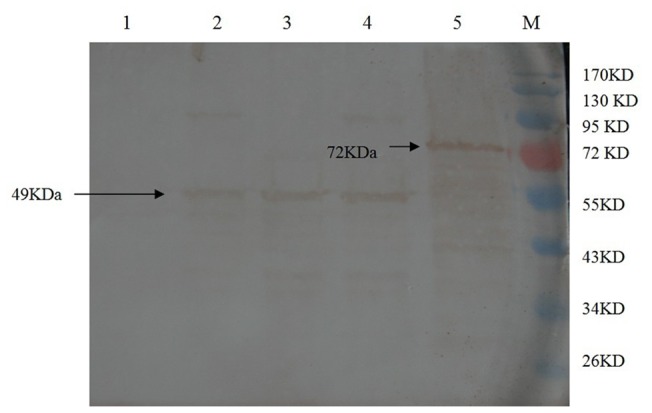
Localization of *Mycoplasma bovis* α-enolase. Western blot analysis of bovine serum albumin (BSA; lane 1), cell soluble cytosolic fraction proteins (lane 2), cell membrane fraction proteins (lane 3), whole cell protein (lane 4), and purified recombinant *Mycoplasma bovis* α-enolase blotted onto a nylon membrane and detected with rabbit anti-recombinant enolase antibodies (lane 5) blotted onto a nylon membrane and detected with rabbit anti-recombinant enolase antibodies. M: protein marker.

MbEno was detected in the cell-soluble cytosolic fraction proteins (Figure 2. lane 2), in the cell-membrane-fraction proteins (Figure 2. lane 3) and in whole-cell proteins (Figure 2. lane 4). Bovine serum albumin (Figure 2. lane 1) and rMbEno (Figure 2. lane 5) were employed as negative and positive controls, respectively. This analysis, using anti-rMbEno antibodies, revealed a strong reactivity to a protein of approximately 49 kDa, suggesting that MbEno is present in both the membrane and the soluble cytosolic protein fractions of *M. bovis* cells.

### M. bovis and rMbEno bind plasminogen

**Figure 3:**
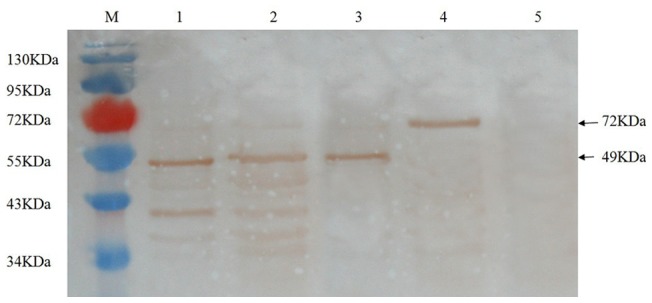
Ligand blotting assay. Membrane fraction proteins(lane 1), soluble cytosolic fraction proteins(lane 2), commercial α-enolase(lane 3), recombinant *Mycoplasma bovis* α-enolase (lane 4), and BSA(lane 5) were blotted onto nitrocellulose membranes following SDS-PAGE, and then incubated with plasminogen post-blocking. Bound plasminogen was detected with sheep anti-plasminogen polyclonal antibody. M: protein marker.

**Figure 4:**
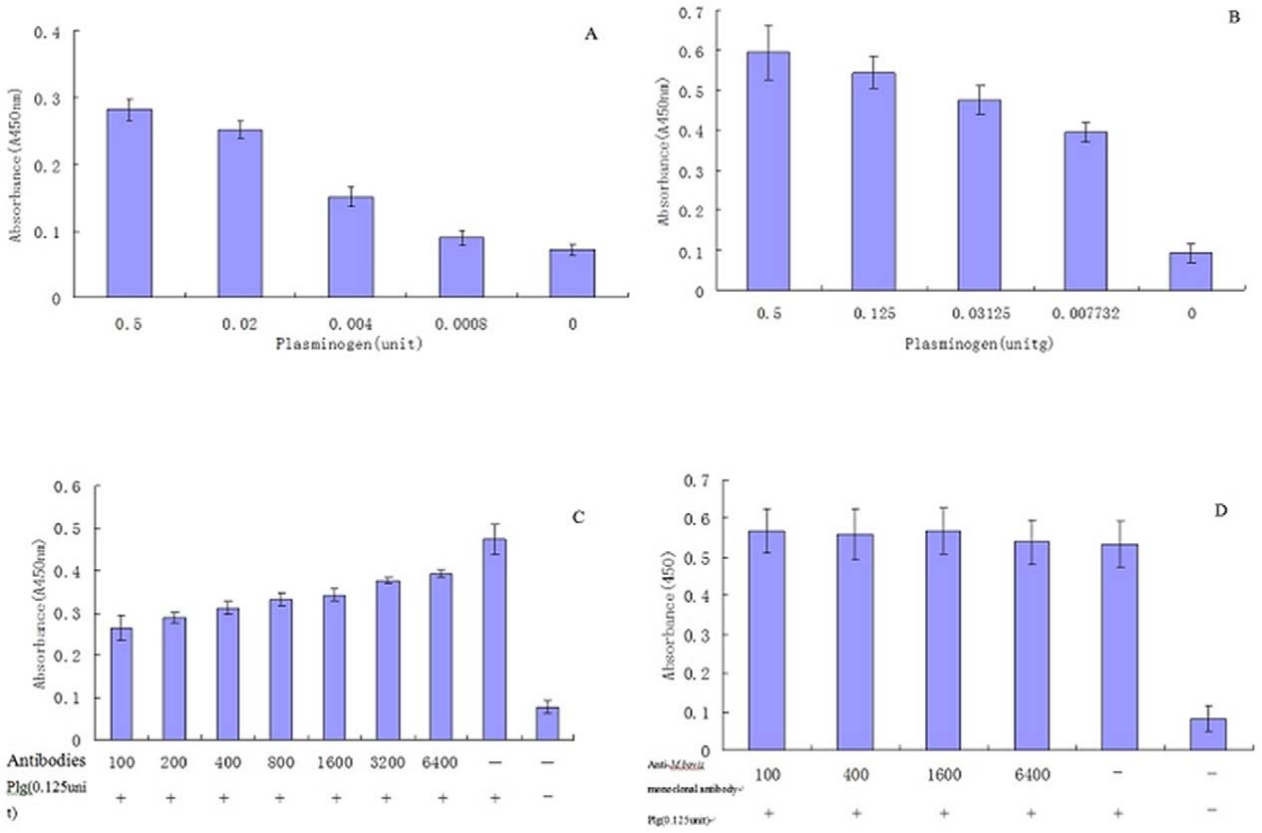
Plasminogen binding assays. Plates were coated as detailed in Materials and Methods.(A) Plasminogen (Plg) (0.5 to 0.007732unit/well) binds to fixed recombinant *Mycoplasma bovis* α-enolase (rMbEno) in a concentration-dependent manner. (B) In a parallel assay, Plg (0.5 to 0.007732/units) binds to whole cell proteins in a concentration-dependent manner. (C) In a competition assay, binding of plasminogen is inhibited by increasing concentrations of anti-rMbEno antiserum (in serial dilutions from 1/100 to 1/6400). (D) Negative control: Plasminogen binding is inhibited by an anti*-M. bovis* monoclonal antibody. Three independent experiments were performed in triplicate. The error bars indicate the standard deviations from three independent experiments.

MbEno was detected among the cell-membrane-fraction proteins (Figure 3. lane 1) and cell-soluble cytosolic-fraction proteins (Figure 3. lane 2). α-Enolase (commercial preparation) (Figure 3. lane3), and rMbEno protein were used as positive controls; BSA was employed as a negative control. We discovered that several *M. bovis* proteins, including α-enolase, interacted with Plg. The ability of rMbEno to bind Plg was tested by ELISA. Increasing concentrations of Plg bound to immobilized rMbEno in a dose dependent fashion (Figure 4A). A similar pattern was observed when the wells were coated with *M. bovis* membrane protein (Figure 4B).

Competition experiments were performed by adding rMbEno rabbit polyclonal antibodies to the system. Addition of increasing concentrations of anti-rMbEno antibodies decreased Plg binding to *M. bovis* in a dose-dependent manner (Figure 4C). In the presence of anti*-M. bovis* monoclonal antibody, however, no obvious effects were observed (Figure 4D).

### Adherence and inhibition assay

**Figure 5:**
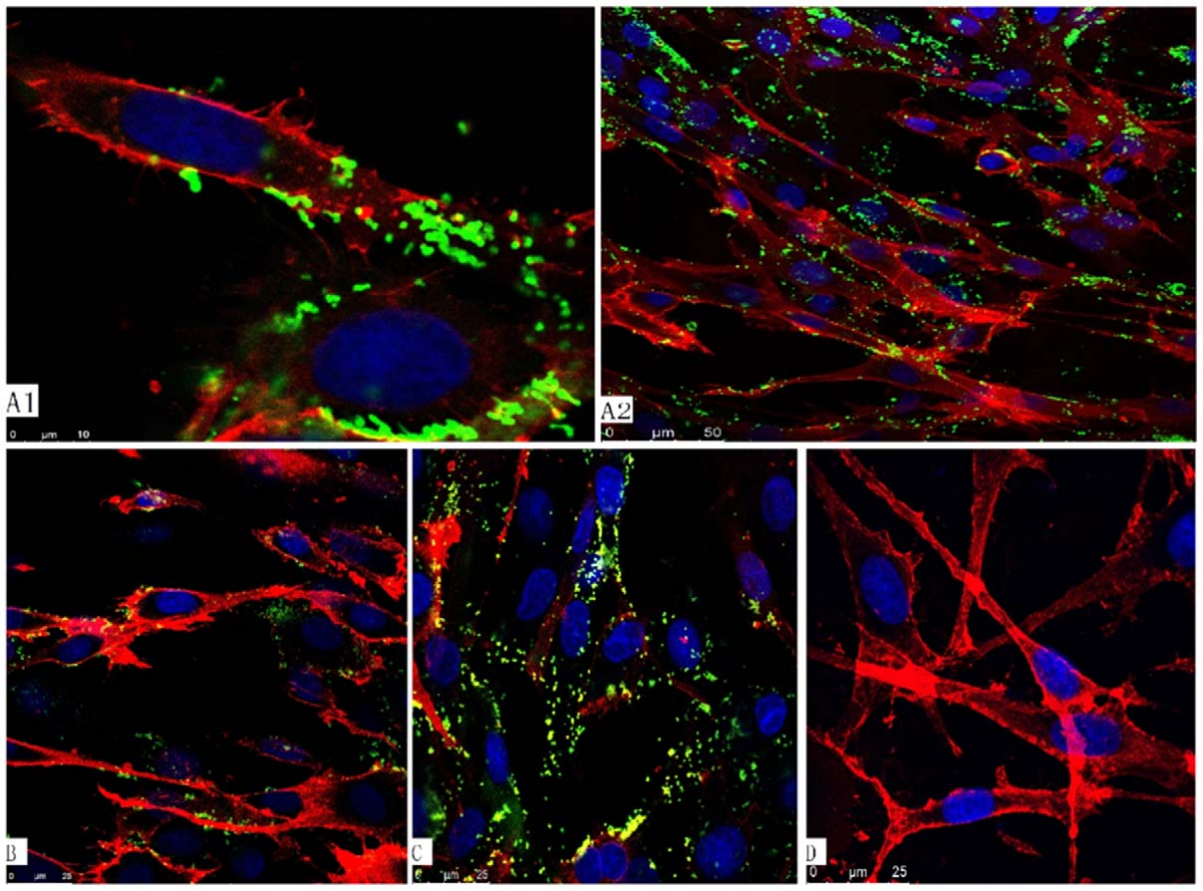
Confocal laser scanning microscopy depicting interactions between the *Mycoplasma bovis* Hubei strain and embryonic bovine lung (EBL) cells. EBL cells prtreated with plasminogen (Plg) were infected for 4 h *M. bovis* (Hubei) (MOI  = 200). The infected melanoma cells were washed, fixed, and immunostained with bovis anti-*M. bovis* antibody and rabbit anti-bovine IgG–FITC. Membranes containing EBL cells were labeled with 1,1′-dioctadecyl-3,3,3′,3′-tetramethylindocarbocyanine perchlorate (DiI)and cell nuclei counter-labeled with 4′,6-diamidino-2-phenylindole (DAPI).(A1–A2) EBL cells prtreated with Plg were infected with the *Mycoplasma bovis* Hubei strain.(B)The adherence process is inhibited by anti-rMbEno antidody(20 ug/ml).(C) In a competition assay, adherence process was inhibited by non-immune rabbit antibodies (20 ug/ml) (D) Uninfected *M. bovis* EBL cell control.

*M. bovis* has been shown to be capable of adhering to EBL cells, and it has been further shown that membrane-protein antibodies can significantly inhibit adherence of the bacterium to such cells. Figure 5 shows the *M. bovis* Hubei strain adhering to EBL cells pre-treated with Plg. We found that adherence to the cells was inhibited by treatment with the anti-rMbEno antibody. In a competition assay, however, we found that infection was not inhibited by treatment with non-immune rabbit antibodies. Furthermore, no fluorescence was detected when EBL cells were incubated with the secondary antibody alone. The results of the confocal laser scanning microscopy (CLSM), therefore, show that α-Enolase is an *M. bovis* adhesion-related factor.

To further validate these experiments, adherence and inhibition values were calculated. Table 1 shows that after pre-incubation with Plg, *M. bovis* EBL cell adherence rates were enhanced by 11.9%. We also noted that proteolysis of the Hubei strain by trypsin resulted in enhanced adherence to EBL cells; this was more effective than that observed with Plg alone.

**Table 1:** Adherence and inhibition assays.

antibodies	Pretreatments		
	HBSS (%)	Plasminogen (0.25unit/ml) (%)	Trypsin (25μg/ml) (%)
Negative control	100	111.9	234.9
Non-immune rabbit antibody	59.4	69.9*	39.7
Rabbit Anti-enolase antibody	57.0	58.8*	37.6

Experiments were performed in triplicate. Hank's Balanced Salt Solution (HBSS). *P<0.05, compared with the corresponding group using non-immune rabbit antibodies.

We observed no obvious difference between the anti-rMbEno antibodies and non-immune rabbit serum in binding inhibition activity tests (P>0.05), because EBL cells were not treated with Plg. The inhibition values also showed no obvious differences between *M. bovis* pretreated with trypsin by anti-rMbEno antibody and the non-immune rabbit serum (P>0.05). However, when the EBL cells were treated with Plg, the anti-enolase antibodies (that inhibited adherence) were more effective than the non-immune rabbit antibodies at inhibiting adherence (P<0.05).

## Discussion

Identification of the complete genomic sequence of *M. bovis* (Hubei strain) α-enolase enabled us to study the α-enolase of this organism. The *M. bovis* α-enolase sequence contains the typical Plg-binding-site motifs that characterize α-enolase proteins. However, *M. bovis* α-enolase lacks classical protein-sorting signals. If the α-enolase is not a surface protein, the function of α-enolase as a Plg-binding protein in *M. bovis* may have less biological significance. However, α-enolase has been shown to be a surface protein in many bacteria species [11], [13], [14]. How α-enolase molecules lacking signal peptides are exported to the cell surface is an unresolved question. Secretion of α-enolase has been found to be SecA2-dependent in *Listeria monocytogenes* [15]; therefore, some researchers have suggested that a similar mechanism may be used to export α-enolase [14]. However, if such a mechanism exists, this presents an important and challenging task that needs to be addressed by further experimentation. Our experiments suggest that *M. bovis* membrane-associated α-enolase is a Plg-binding protein.

In this study, we have provided evidence that the α-enolase is present in both the soluble cytosolic fraction and the membrane fraction of *M*. *bovis*; a similar finding has been made in *M. fermentans* using transmission electron microscopy [10]. **Figure** 2(lanes 2 and 3) show the presence of MbEno in both the membrane and soluble cytosolic protein fractions of *M. bovis*. This analysis demonstrates that α-enolase is a membrane-associated protein in *M. bovis*, as has been found in many other bacterial species [9]–[12]. We suggest that the presence of α-enolase in this location is consistent with the etiology of adherence to host cells.

That α-enolase plays a role in the invasion of host tissue by pathogens exhibiting adhesion has already been demonstrated [13]. Recent studies indicated that α-enolase on the cell surface of *Streptococcus pneumoniae* and *M. fermentans* binds and activates plasminogen [10], [16] and influences *Streptococcus pneumoniae* adherence to human pharyngeal cells [16]. α-enolase confers surface-associated proteolytic activity, which may facilitate pathogen invasion and dissemination in the infected host [9].

Interestingly, western immunoblot assays revealed that several different proteins within the *M. bovis* membrane protein fraction bound to Plg. Figure 4 shows that the rabbit anti-rMbeno antibody inhibits the binding of Plg to *M. bovis* whole-cell proteins, supporting our hypothesis that α-enolase is a major Plg-binding protein in *M. bovis*.

Our research supports the hypothesis that the *M. bovis* α-enolase is a Plg-binding protein, and we speculate that α-enolase is involved in *M. bovis* adhesion to EBL cells. The triple immunofluorescence labeling technique used here, when combined with CLSM, offers a visual way to differentiate between adherence and adherence inhibition in mycoplasmas. Utilization of CLSM revealed that the degree of *M. bovis* adherence to the cell surface of EBL cells is related to the availability of Plg and α-enolase.

The adherence and inhibition assay results confirm that Plg facilitates the binding of *M. bovis* to EBL cells. Surprisingly, proteolysis of *M. bovis* by trypsin resulted in significantly enhanced binding to EBL cells in comparison with Plg alone, and the enhancement was not specifically inhibited by the addition of anti-rMbeno antibody. This result suggests that other protease-binding regions in the *M. bovis* cell membrane are involved in proteolysis, as has been described previously [17], and that these regions enhance *Mycoplasma* adherence to host cells. Some research has shown that proteases such as trypsin and proteinase K reduced mycoplasma binding to cells by hydrolyzing the surface adhesion-related protein [18]. We believe that the presence of large numbers of proteases would reduce binding by hydrolysis of surface proteins; however, in the presence of trace amounts of protease, the protease would enhance binding by giving mycoplasmas proteolytic activity. Essentially, whether protease pretreatment makes mycoplasmas more effective at binding cells is probably dependent on the concentration of the protease itself and the reaction conditions present at the time.

α-enolase has been detected in studies attempting to identify proteins interacting with host surfaces in bacteria [12], [16]. To functionally characterize the *M. bovis* α-enolase, both the recombinant and the native proteins were subjected to an *in vitro* plasminogen-binding assay. In this work we have demonstrated the ability of *M. bovis* α-enolase to bind Plg; this could be of great importance for *Mycoplasma* establishment in the host, allowing adhesion to the EBL cells. Other studies have shown that α-enolase is not only an adhesion-related factor, but also an auto-antigen in connective tissue diseases [19]. We speculate that α-enolase may be involved in various clinical and pathologic sequelae of *M. bovis* infection, such as arthritis, tenosynovitis and meningitis.

### Conclusions

In conclusion, our studies show that the α-enolase of *M. bovis* is a surface-exposed protein that enables *M. bovis* adherence to EBL cells by binding Plg. Howere, several other Plg receptors exist on the *M. bovis* cell surface. Our finding that trypsin enhances adherence more effectively than does Plg is noteworthy and may provide some insight towards identifying the mechanisms underlying *M. bovis* adherence to host cells.

## Materials and Methods

### Ethics Statement

Animal experiments were approved by Harbin Veterinary Research Institute of the Chinese Academy of Agricultural Sciences and National Engineering Research Center of Veterinary Biologics. And animal experiments were performed in accordance with animal ethics guidelines and approved protocols. The Animal Ethics Committee approval number was Heilongjiang-SYXK 2006-032.

### Bacterial strains, cell lines, and culture conditions

The *M. bovis* strain Hubei isolated in China [6], was used in this study. In these experiments, mycoplasmas were grown in modified pleuropneumonia-like organism (PPLO) medium containing 20% donor equine serum(Hyclone, Logan, USA), 10% yeast extract, thallium acetate (0.125 mg/ml) and penicillin (200 IU/ml) [20].

The EBL cells [21] were kindly provided by Pro. Xue (National Engineering Research Center of Veterinary Biologics, Harbin, China).

### Cloning, M. bovis α-enolase gene expression, and protein purification

The α-enolase ORF was identified from the *M. bovis* strain Hubei strain complete genome sequence [22]. The *M. bovis* α-enolase ORF containing two TGA codons, which were *in vitro* terminators, encoded tryptophan in *Mycoplasma*. Primers eno1/eno2, eno3/eno4 and eno5/eno6 (Table 2) were designed for site-directed mutagenesis and used in overlapping PCRs. The full-length mutated gene was expressed by pET System (Novagen, Madison, USA). The resulting rMbEno protein was purified using HisPur Ni-NTA Resin and Kits (Thermo, Rockford, USA).

**Table 2:** Overlapping PCR primers using for amplification of α-Enolase.

Primers name	Sequence (5′→3′)	Localization (nt)
Eno1 Eno2 Eno3 Eno4 Eno5 Eno6	cag gga tcc atg cct att att gaa aca att caa g tca taa cac ctttgc cgc cga acc agt ttc ctt cggc cgc aaa ggt gtt atg aca gcc gtt gat aat gtt a tcg ctt tca gca aga cca tct tca att gaa ata ata gga t tct tgc tga aag cga ttg gga agg att tgc aaa aat gac t cgg gtc gac tta ctt tat gtt gta aaa tgc ttt aga acc	1–186 186–960 960–1365

### Antibody production

Polyclonal antibodies against *M. bovis* α-enolase were prepared in female New Zealand White rabbits by injection with rMbEno protein. The rabbits were bled ten days after the third immunization and the antibody titers measured by ELISA. Antibodies were purified using Protein A High-Capacity Agarose and Kits (Thermo) and quantified using a BCA Protein Assay Kit(Beyotime Institute of Biotechnology, Nan Tong, China).

### Preparation of M. bovis protein fractions

Membrane and cytosolic protein fractions from *M. bovis* were obtained using ProteoExtract Transmembrane Protein Extraction Kit (Novagen) according to the manufacturer's instructions. Whole cell protein extracts were prepared by sonication. Protein quantitation was performed with the BCA Protein Assay Kit.

### Cellular localization of M. bovis α-enolase

To locate the cellular distribution of MbEno, Western immunoblot assays were performed as described previously [14]. Briefly, the gels were transferred to nitrocellulose membranes (PALL, Ann Arbor, USA) and blocked with 5% gelatin derived from cold-water fish skin (Sigma). Following three washes with PBST (PBS containing 0.05% Tween-20), nitrocellulose(NC) membrane was incubated with anti-rMbEno antibody(1∶800). Bound antibodies were detected by incubation with anti-rabbit IgG (whole-molecule) peroxidase conjugate (Sigma). Cross reacting protein bands were visualized using a DAB Substrate Kit (Thermo).

### M. bovis α-enolase plasminogen binding assays

A ligand blotting assay was performed to test the ability of the *M. bovis* α-enolase to bind plasminogen, as described previously [12]. Briefly, *M. bovis* fractionated proteins and rMbEno were transferred onto NC membranes, and incubated with Plg (Sigma). Blots were developed with DAB Substrate Kit.

ELISA was performed to verify the ability of the proteins to bind Plg [14]. ELISA plate wells were coated with rMbEno. In a parallel experiment, ELISA wells were coated with membrane protein fraction from *M. bovis* cells and a range of concentrations of Plg added to the wells. The inhibition experiment was performed by adding anti-rMbEno antibody (in serial dilutions from 1/100 to 1/6,400) prior to the addition of Plg. A set of competition experiments were also performed that included the addition of anti*-M. bovis* monoclonal antibody (Abcam, Hong Kong, China). Protein-protein interactions were identified through use of sheep anti-plasminogen polyclonal antibodies. The binding was determined by incubation with anti-sheep antibodies. The absorbance was measured at A450 using a microplate reader (Bio-Tek Instruments, Winooski, USA).

### Adherence and inhibition assay of M. bovis

Invasion of *M. bovis* strain Huibei was determined using CLSM [23]. After washing to remove non-adherent mycoplasmas, *M. bovis*-infected EBL cells were fixed with 4% paraformaldehyde (PFA). The dish containing the cells was overlaid with anti-*M. bovis* antiserum. Antibodies bound to the cells were detected by rabbit anti-bovine IgG (whole molecule)–FITC (Sigma). The membranes containing EBL cells were labeled with 1,1′-dioctadecyl-3,3,3′,3′-tetramethylindocarbocyanine perchlorate (DiI). Cell nuclei were labeled with 4′,6-diamidino-2-phenylindole (DAPI). Immunofluorescent was evaluated using a Leica TCS SP5 laser scanning confocal microscope (Leica, Mannheim, Germany).

Adherence and inhibition of adherence was determined using a bacteriological assay, as described previously [24]. Plg(0.25 units/ml) was incubated with EBL cells. The cells then were infected with *M. bovis* (Hubei) at a multiplicity of infection (MOI) of 200. Non-adherent mycoplasmas were removed by washing. The cells were lysed with 0.25% trypsin (Gibco) and serial dilutions of the cell lysate were plated onto solid modified PPLO medium. The *Mycoplasma* colonies were counted to determine the adherence frequencies. For the adherence inhibition assay, *M. bovis* (Hubei) was incubated with rabbit antibody raised against rMbEno (20 μg/ml), or non-immune rabbit antibody (20 μg/ml) prior to infecting the EBL cells (as described above). And a parallel experiment was performed by replacing the native *M. bovis* preparation in the adherence reaction mixture with *M. bovis* that had been pre-incubated with trypsin (25 μg/ml) for 15 min at 37°C (all steps thereafter were performed as described above). The percent inhibition was calculated using the following formula: (CFU each cell treated with rabbit antibody/CFU each untreated cell) ×100.
